# Brain Expressed and X-Linked (Bex) Proteins Are Intrinsically Disordered Proteins (IDPs) and Form New Signaling Hubs

**DOI:** 10.1371/journal.pone.0117206

**Published:** 2015-01-22

**Authors:** Eva M. Fernandez, María D. Díaz-Ceso, Marçal Vilar

**Affiliations:** Laboratory of Neurodegeneration, Chronic Disease Program (UFIEC), Spanish Institute of Health Carlos III (ISCIII), Majadahonda, Madrid, Spain; Russian Academy of Sciences, Institute for Biological Instrumentation, RUSSIAN FEDERATION

## Abstract

Intrinsically disordered proteins (IDPs) are abundant in complex organisms. Due to their promiscuous nature and their ability to adopt several conformations IDPs constitute important points of network regulation. The family of Brain Expressed and X-linked (Bex) proteins consists of 5 members in humans (Bex1-5). Recent reports have implicated Bex proteins in transcriptional regulation and signaling pathways involved in neurodegeneration, cancer, cell cycle and tumor growth. However, structural and biophysical data for this protein family is almost non-existent. We used bioinformatics analyses to show that Bex proteins contain long regions of intrinsic disorder which are conserved across all members. Moreover, we confirmed the intrinsic disorder by circular dichroism spectroscopy of Bex1 after expression and purification in *E. coli*. These observations strongly suggest that Bex proteins constitute a new group of IDPs. Based on these findings, together with the demonstrated promiscuity of Bex proteins and their involvement in different signaling pathways, we propose that Bex family members play important roles in the formation of protein network hubs.

## Introduction

**Figure 1:**
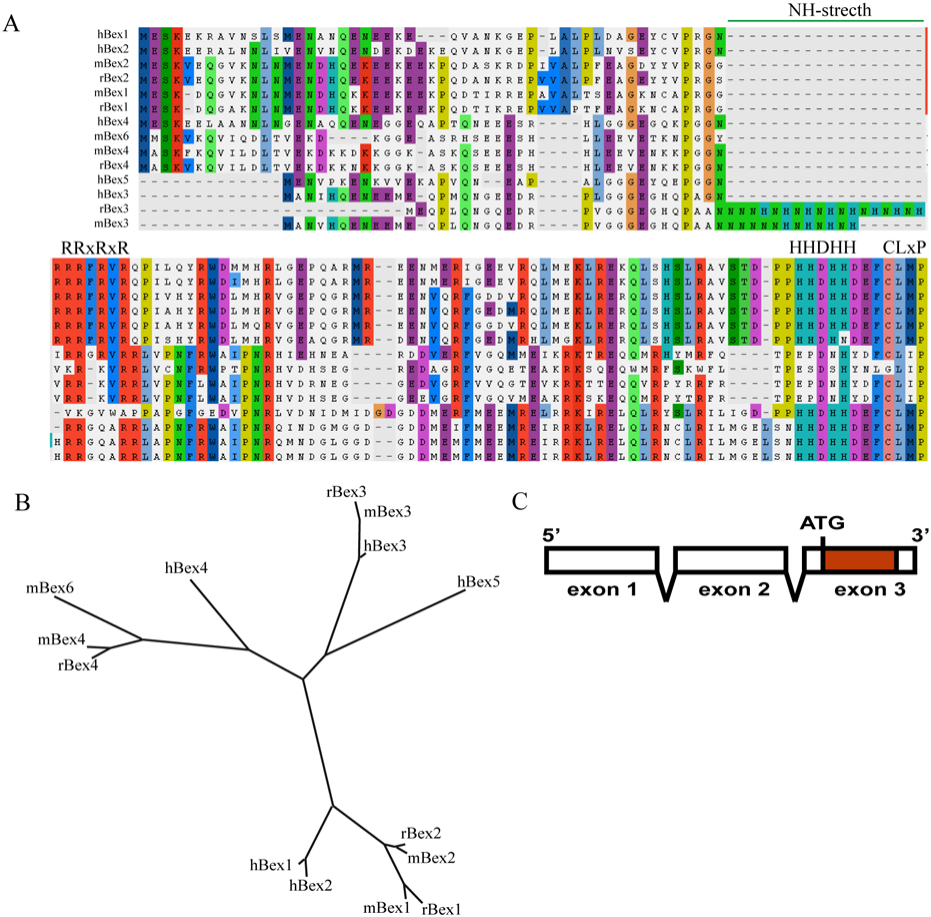
Sequence alignment and phylogenetic analysis of human, rat and mouse Bex proteins. A) Protein alignments of human (h), rat (r), and mouse (m) Bex proteins as determined using CLUSTAL-W2 software. Conserved residues are highlighted in colors. The accession number of the Bex protein sequences analyzed in human (h), mouse (m) and rat (r) are hBex1 (NP_060946.3), hBex2 (NP_116010.1), hBex3 (AAX40680.1), hBex4 (NP_001121160.1), hBex5 (NP_001153032.1), mBEX1 (NP_033078.2), mBEX2 (NP_033879.1), mBEX3 (NP_001103703.1), mBex4 (NP_997622.1), mBex6 (NP_001028711.1), rBex1(NP_001032442.1), rBex2 (NP_001070903.1), rBex3 (NP_445853.1) and rBex4 (XP_003754868.1). Histidine and asparagine-rich regions (NH-stretch), the RRxRxR motif and the CLxP motif are highlighted. B) Phylogenetic tree derived from the alignment sequence. Bex proteins are classified into three different subgroups. C) Conserved gene structure of all Bex proteins. The ORF coding region is located in the third exon (red).

The Bex (brain-expressed X-linked) gene family is a recent acquisition in the mammalian lineage, and no orthologues have to date been described in non-mammalian species [1]. In humans, the Bex family consists of five members (Bex1–5). Rodents lack Bex5, but an additional member (mBex6) is found exclusively in mice [2]. All Bex family members have three exons, the third of which encodes the full protein (Fig. 1). In humans all five members of the Bex family are encoded on the X chromosome in tandem (in the Xq22 region in humans). Bex5, Bex1 and Bex2 are located on the negative strand and Bex4 and Bex3 on the positive strand [1–3]. This physical arrangement is highly conserved in all species studied [3]. Bex1 and Bex2 share similar expression patterns in the central nervous system, with high levels in the pituitary, cerebellum, and temporal lobe [2]. Bex1 is expressed in other tissues at levels similar to those found in the brain. Bex4 is highly expressed in the heart and skeletal muscle [2], while in human Bex3 and Bex5 are ubiquitously expressed (Bex3 at higher levels than Bex5) [2].

While the precise function of Bex proteins remains unclear, they are known to participate in signaling pathways involved in apoptosis, cancer, gene regulation and the cell cycle and are candidate tumor suppressor genes [4–6]. The Bex1 gene was initially cloned in blastocysts in preimplantation stage mouse embryos [7], and is expressed very early in embryonic development [8]. Bex proteins have been implicated in apoptosis/survival pathways in different cells of tumor origin. The subcellular localization of Bex proteins has been described [2], and ranges from predominantly nuclear (Bex1) to predominantly cytoplasmic (Bex3, Bex5 and Bex6), with some proteins found in both the nucleus and cytoplasm (Bex2 and Bex4) [2, 9]. This subcellular localization pattern in the cytoplasm and nucleus suggests potential roles of Bex proteins as gene regulators.

Intrinsically disordered proteins (IDPs) lack a unique 3D structure: due to their highly dynamic nature, they possess no single defined structure, but instead exist as a dynamic ensemble of conformations (extensively reviewed in [10]). Computational analysis suggests that eukaryotes have a higher number of IDPs than prokaryotes [11, 12]. This may be due to the greater complexity of eukaryotic signaling and regulatory pathways, which require the presence of disordered regions. It is widely accepted that the lack of a “static” or “fixed” structure in IDPs confers several advantages over folded proteins as regards promiscuous protein interactions, such as high specificity with lower affinity, and the ability to recognize multiple binding partners with distinct interaction surfaces [13–17]. Owing to these properties, IDPs are abundant in many signaling pathways, and include several transcription factors that form hubs in signaling networks [18, 19]. The identification of IDPs has been made possible by the development of powerful *in silico* computational methods that predict the presence of disordered regions with protein sequences [20–23]. This bioinformatic approach can be complemented with biochemical and biophysical analyses [13].

Although Bex proteins were described some years ago, the literature offers little in the way of structural data for this protein family. Bex genes encode small proteins of 100-130 residues, with no known conserved functional domains within their sequences. A better understanding of the structural properties of these proteins could provide clues as to their functional roles within the cell. In the present study, we predicted the presence of disordered regions in Bex proteins and experimentally corroborated our findings by circular dichroism. Based on our findings, we propose that Bex proteins constitute a new group of intrinsically disordered proteins (IDPs) and participate in the formation of new protein network hubs.

## Materials and Methods

### Protein Sequences and protein alignment

The accession numbers of the Bex protein sequences analyzed in human (h), mouse (m) and rat (r) were as follows: hBex1 (NP_060946.3), hBex2 (NP_116010.1), hBex3 (AAX40680.1), hBex4 (NP_001121160.1), hBex5 (NP_001153032.1), mBex1 (NP_033078.2), mBEX2 (NP_033879.1), mBex3 (NP_001103703.1), mBex4 (NP_997622.1), mBex6 (NP_001028711.1), rBex1(NP_001032442.1), rBex2 (NP_001070903.1), rBex3 (NP_445853.1) and rBex4 (XP_003754868.1). Protein alignment was performed with CLUSTAL-W2 (http://www.ebi.ac.uk/Tools/msa/clustalw2), using default settings.

### Prediction of disordered regions and coiled coils

Predictions of disorder in the human Bex1–5 and mouse Bex6 proteins were made using several software predictors: the DisProt server (http://www.disprot.org/pondr-fit.php), which provides several different software tools to predict protein disorder (PONDR-FIT [24], VL3, VSL2, and VLTX [25]); RONN [26]; DISOPRED3 [27]; Pr-DOS [28] and DisPSSMP [29]. Hydrophobic cluster analysis (HCA) [30] was performed at SA-Search v1.0 (http://bioserv.rpbs.jussieu.fr/RPBS/cgi-bin/). Coiled-coil regions were predicted using the following servers: MARCOIL [31], MULTICOIL [32], PCOILS and COILS [33, 34], using default settings. ANCHOR and IUPRED predictions were made using the ANCHOR server (http://anchor.enzim.hu/pred.php).

### Cloning, expression and purification of mBex1 in *E. coli*

Mouse Bex1 expression constructs were generated as glutathione-s-transferase (GST) or histidine tags at the N-terminal in pGEX6P (GE-Healthcare Life Sciences) or pET28 (Novagen) plasmids, respectively. Unfortunately, those constructs give rise to a cleaved fusion protein. We thus created a new expression construct with untagged mBex1 in pET28a (Novagen) using NcoI and XhoI cloning sites. This cloning strategy removes the histidine tag from the pET28a and Bex1 is expressed using its own starting methionine residue. The mBex1 sequence was amplified from pcDNA3-Bex1 [9] and ligated in pET28a after digesting both the PCR product and plasmid with NcoI and Xho I restriction enzymes. The construct was verified by DNA sequencing using local genomic services from Instituto de Salud Carlos III. Protein expression and purification was carried out following a previously described method based on the heat resistant nature of the IDPs [35]. Briefly, proteins were expressed in BL21(DE3) *E. coli* (New England Biolabs), which was grown in Luria broth medium containing 50 μg/ml kanamycin (SIGMA) at 37°C until reaching an OD600 reading of 0.6. The cultures were then induced with 0.5 mM IPTG (Apollo Scientific), incubated at 37°C for 3 hours, and finally harvested by centrifugation at 6000 × g for 15 min at 4°C. Cell pellets (collected from 500 ml of culture) were suspended in 25 ml of water. The cells were boiled for 5 minutes and placed on ice for 5 minutes. The suspension was centrifuged for 15 minutes at 15,000 × g and 4°C. SDS-PAGE of the supernatant identified Bex1 as an 80% pure protein. The supernatant was then filtered through a 0.22-μm polyvinylidene difluoride (PVDF) membrane (Millipore) in preparation for chromatography. NaCl was added to the supernatant at a final concentration of 3 M, and the solution was loaded into a hydrophobic HiButyl-S-Sepharose chromatography column (GE-Healthcare Life Sciences) and eluted with 20 mM acetic acid/sodium acetate pH 5.1 using a chromatography system (GE-Healthcare Life Sciences). A high peak corresponding to nucleic acid appeared in the initial fractions followed by the elution of mBex1. mBex1 fractions were dialyzed against 20 mM acetic acid/sodium acetate [pH 5.1] and analyzed by circular dichroism.

### Circular dichroism (CD)

CD spectra were recorded at 20°C on a Jasco 810 spectropolarimeter module equipped with a temperature-control system. Data were collected using the Jasco software package. Proteins were prepared in 20 mM acetate buffer pH 5.1 at a concentration of 20 μM. CD spectra were measured between 195 and 260 nm in a 1-mm cuvette and averaged over 5 scans. The experimental spectra were adjusted to subtract the spectrum of the buffer. The mean residue ellipticity (MRE) was calculated using the following equation: MRE = molecular weight [Da]/(number of residues – 1). The mean ellipticity values [θ] were calculated as follows: [θ] = (millidegrees value × MRW)/(pathlength [mm] × concentration [mg/ml]). Experimental data in the 195-240 nm range were analyzed using the K2D3 online CD server (http://k2d3.ogic.ca//index.html) [36].

## Results

### Alignment of the protein sequence of Bex proteins

The alignment of Bex proteins from human, mouse and rat is shown in Fig. 1A. Bex proteins are generally small (100-130 residues in length). The amino acid identity shared by Bex protein pairs is over 90% for hBex1 and hBex2; 44% for hBex1 and hBex3; 39% for hBex1 and hBex4; and 44% for hBex1 and hBex5. Similar identity percentages have been reported in all species analyzed to date [3]. A notable sequence feature that has been previously described [7, 37] is the presence of the CaaX motif at the very end of the C-terminus in all Bex proteins. This sequence has been described as a prenylation motif to target Ras to the plasma membrane [38, 39], although the function of this region in Bex remains unknown. All Bex members except for Bex4 share a conserved stretch of histidine residues at the C-terminus that constitute a putative metal binding region (Fig. 1A). Bex3 has been reported to bind cobalt [2]. rBex3 also contains an internal region that is very rich in histidines and asparagines and includes 7 alternating repeats of histidine and asparagine (Fig. 1A). By contrast, mBex3 has five repeats and hBex3 has no repeats. The role of this region in the function of Bex3 remains unclear. Bex1 and Bex2, but not Bex3, Bex4 or Bex6, have a highly conserved, arginine-rich region (R-rich region), RRxRxR, in the protein sequence. This motif constitutes a functional nuclear localization sequence (NLS), as mutation to alanine impairs nuclear localization [37, 40]. The phylogenetic tree of the Bex protein alignment (Fig. 1B) shows three separate sub-groups consisting of Bex1 and Bex2, Bex3 and Bex5, and Bex4 and mBex6.

### Bex3 and Bex5 contain a coiled-coil domain

Due to the paucity of structural studies of the Bex protein family, we began by making secondary structure predictions using the PSIPRED server (http://bioinf.cs.ucl.ac.uk/psipred/)[41]. The PSIPRED v3.3 software tool [42] predicted the existence of secondary structure elements (in the form of α-helices) in all Bex proteins, with some differences in the location and the extent of the α-helix (S1–S5 Figs.). Four helices were predicted for Bex1 and Bex2, which share very similar protein sequences (S1 and S2 Figs.), two for Bex3 and Bex5 (S3 and S4 Figs.) and two for Bex4 (S5 Fig.). In all Bex proteins the presence of a long α-helix was predicted in the region between residues 60 and 90, with the remainder of the protein sequence in a random coil conformation, suggesting that most of the protein sequence is disordered.

**Figure 2:**
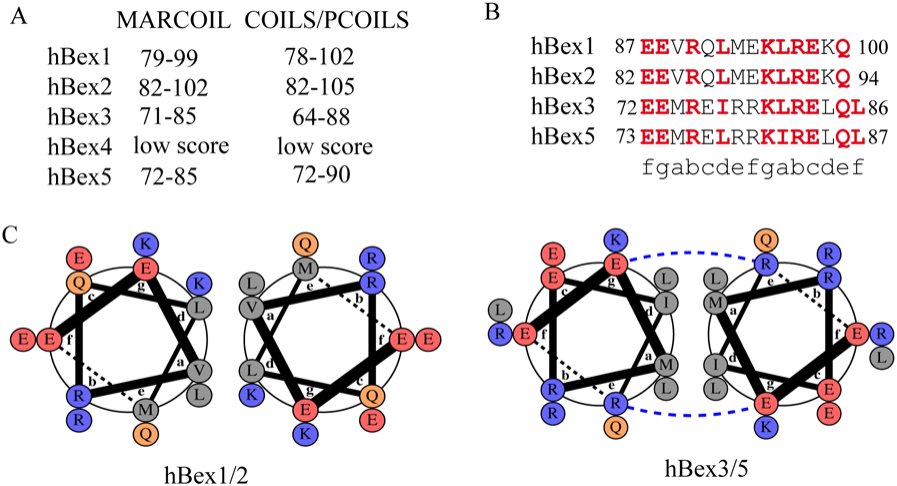
Prediction of coiled-coil regions in Bex1–5. A) Summary of predicted coiled-coil regions in Bex proteins using different servers (see text for details). All regions listed are predicted with a score >0.9. The location of the predicted coiled coil is conserved in Bex1, 2, 3 and 5. No coiled coils are predicted in hBex4. B) Protein sequence alignment of the coiled coils predicted in A, showing the central residues with the highest score predictions. Conserved residues are highlighted in red. The position of each residue in the heptad of the coiled coil is indicated beneath the sequences. C, D) Models of the homodimer interaction mediated by the coiled coils in hBex1,2 (C) and hBex3,5 (D). Electrostatic stabilization interactions between monomers are indicated with dashed blue lines and are only predicted for hBex3,5.

Coiled-coil domains are disordered in a monomeric form but become folded upon formation of oligomers. To investigate the possibility that Bex proteins contain coiled-coil domains we used four coiled-coil predictor programs: MARCOIL [31], MULTICOIL [32], PCOILS and COILS [33, 34] (Fig. 2A). The consensus result from all predictions indicated a high probability of a coiled-coil domain close to the C-terminal in hBex1–3 and hBex5 (at residues 80-100), but not in either hBex4 or mBex6. The alignment of the coiled-coil regions predicted for hBex1–3 and hBex5 is shown in Fig. 2B. Some experimental data suggest the presence of coiled coils in Bex proteins. Bex3 homodimerization has been repeatedly reported in the literature, even in denaturing SDS-PAGE and in reducing conditions [43–45]. The homodimerization domain has been identified in the region comprising residues 81–112 [45], a location that closely matches that of our predicted coiled coil. We modeled the homodimers mediated by the coiled coils as parallel homodimers (Fig. 2C). In the heptad of a coiled coil specific positions are required to maintain the homodimer conformation [46]. The structure is maintained by two different types of interactions: i) electrostatic interactions between the “e” and “g” positions (R79 and E73 in hBex3) and by ii) hydrophobic interactions between the “a” and “d” positions (M74 and I77; L81 and L84 in hBex3) of the heptad (Fig. 2C). The Bex3 model suggests strong stabilization of the coiled coil. Experimental data has shown that mutation of the residues L94/L97/L99 to alanine interferes with homodimerization [45]. The L84 (L81 in hBex3) and L97 (L84 in hBex3) residues constitute the hydrophobic residues in positions “a” and “d” of the predicted coiled coil (Fig. 2B). Based on these data we propose that Bex3 contains a functional coiled-coil domain, which mediates Bex3 dimerization. Dimerization has not to date been described for Bex1, Bex2 or Bex5. We predict that if such homodimers exist, they would be less stable as they lack both the electrostatic stabilization in the “e” and “g” positions and the strong hydrophobic core (Fig. 2C). For Bex5, we predict the formation of a homodimer that is as stable as that of Bex3 (Fig. 2C). Further structural studies of these domains will be necessary to rule out the possible formation of higher order complexes, such as trimers or tetramers, via coiled coils.

### Intrinsic disorder predictions for Bex proteins

Because the aforementioned predictions only revealed small regions with secondary structure, different servers were used to predict the presence of disordered regions in the Bex protein family. Several disorder predictors have been developed in the last decade, each differing in the extent (*i.e.*, the amount of residual secondary and/or tertiary structure) and length of disorder predicted [20, 23]. Since different predictors rely on different physicochemical parameters [20, 21, 23], a given predictor may better detect a particular feature of a disordered protein. Thus, in order to decipher the modular organization of a protein, it is necessary to combine multiple predictors [20, 21, 23]. For our analysis we used 8 different disorder predictors. From the DisProt server [24] we used several tools, including PONDR-Fit [24], VSL2 [47], VL3 [47] and VLXT [25]. We extended our analysis using DisPSSMP [29], DISOPRED-3 [48], Pr-DOS [28] and RONN [26].

**Figure 3:**
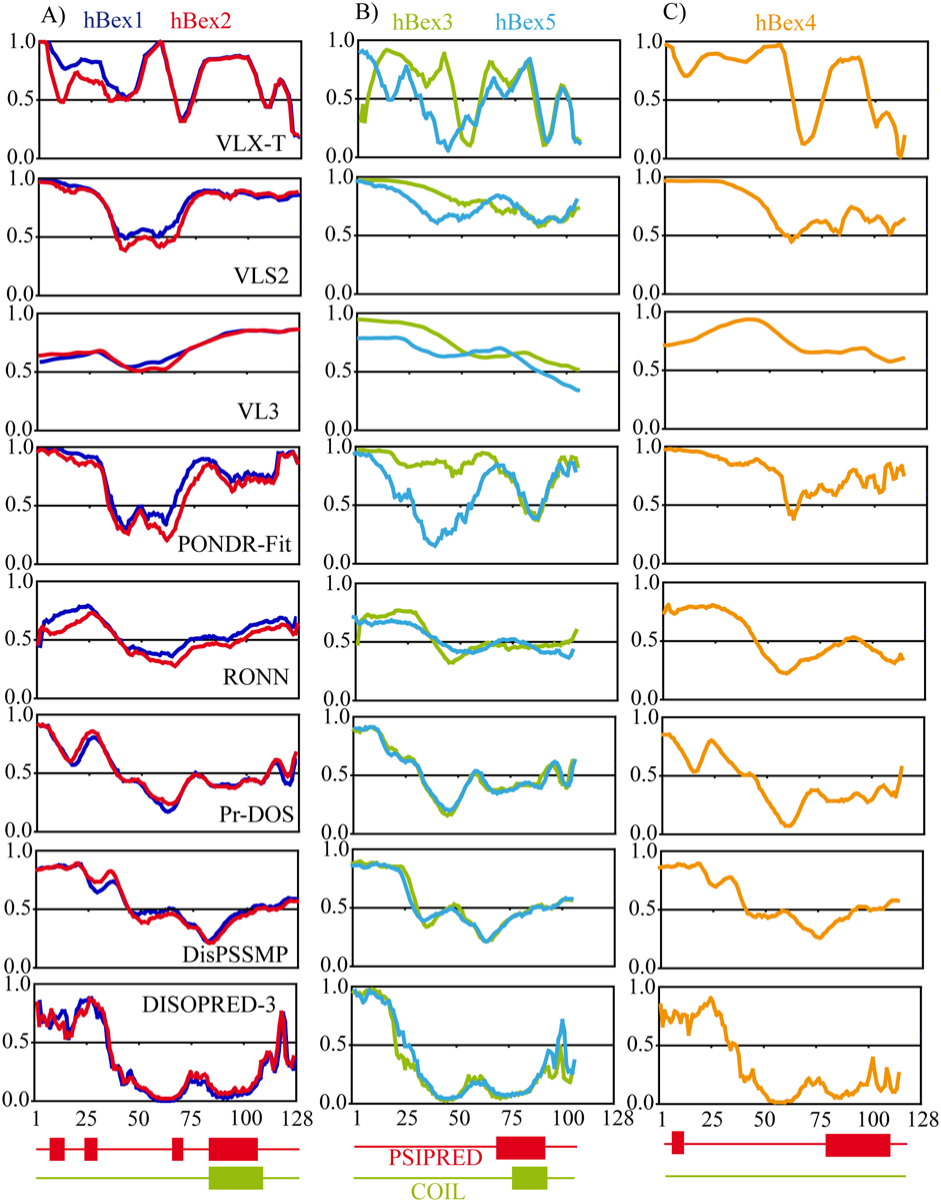
Disorder predictions for the Bex protein family in human: A) Bex1 and Bex2; B) Bex3 and Bex5; C) Bex4. Disorder predictions are sorted from top to bottom according to decreasing average disorder tendency in the region of the coiled coil as predicted using COIL (green box). The location of the α-helix predicted by PSIPRED is shown in red. Disordered regions have scores >0.5.

Comparison of the different outputs from these servers revealed several different profiles (Fig. 3). We organized the output into three groups according to sequence homology consensus: Bex1 and Bex2 (Fig. 3A); Bex3 and Bex5 (Fig. 3B); and Bex4 (Fig. 3C). It should be noted that the presence of coiled-coil regions causes different servers to predict these regions as either ordered or disordered [21]. This effect was also observed in our analysis of Bex proteins. The results obtained using different prediction tools are listed in order of increasing predicted order (top to bottom) in the coiled-coil region (Fig. 3). VLXT, VSL2, VL3 and PONDR-Fit predicted that the coiled-coil region was highly disordered, while DISOPRED-3 and DisPSSMP predicted a high level of order. Other servers such as RONN and PrDOS produced ambiguous predictions (order/disorder). A similar profile was observed for all Bex proteins analyzed (Fig. 3). The presence of coiled coils has been reported to cause discrepancies between different predictors for certain proteins, such as heat shock factor binding protein 1 (HSBP1) [21]. In addition to the coiled-coil region, the consensus prediction indicated a disordered region in the N terminus of up to 50 residues in length, another in the final 20-30 residues of the C terminus, and a third region with secondary structure between residues 80 and 100.

**Figure 4:**
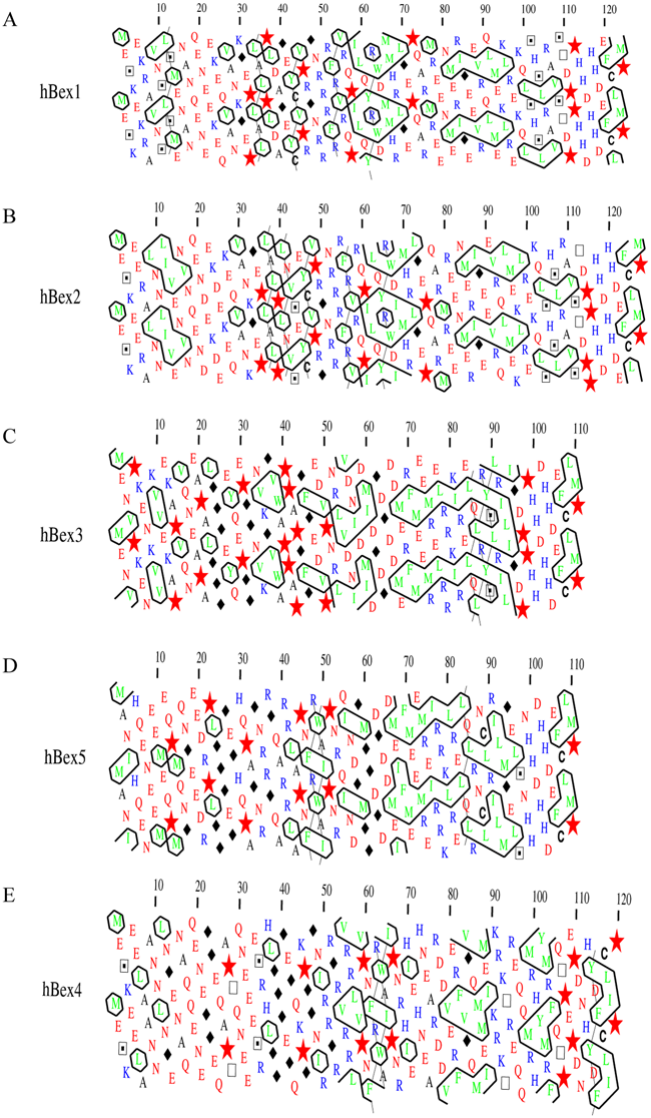
HCA analysis of all human Bex proteins. Bex1 (A), Bex2 (B), Bex3 (C), Bex5 (D) and Bex4 (E) were analyzed using the HCA server (http://bioserv.impmc.jussieu.fr/). The form of the clusters is generally indicative of the type of secondary structure formed (vertical clusters often correspond to β-strands while horizontal clusters correspond to α-helices) [30]. Symbols are used to denote specific amino acids: star for proline; square for threonine; dotted square for serine; diamond for glycine.

We next used hydrophobic cluster analysis (HCA) to better define the boundaries of the disordered regions. HCA provides a 2D helical representation of protein sequences in which hydrophobic clusters are plotted along the sequence [30]. Disordered regions are recognizable as they exhibit reduced numbers of hydrophobic clusters, or none at all. HCA also provides additional, qualitative information as compared with automated predictors. In particular, HCA can identify regions with a coiled coil, regions with a biased composition, and regions with potential for induced folding, and enables better definition of the boundaries of disordered regions [30]. Analysis of the HCA profiles of hBex1–5 revealed several important findings, as shown in Fig. 4. First, all Bex proteins contained a region that was largely devoid of hydrophobic residues and rich in N, E, Q and D residues. This region was putatively disordered, in agreement with the results of the disorder prediction servers, and was located at residues 15-25 for Bex1 and Bex2, 2–20 for Bex3 and Bex5, and 1–30 for Bex4. Second, a region rich in P residues (red stars in the Fig. 4) was identified, mainly concentrated between residues 30 and 50 in Bex1 and Bex2, and at residues 50–70 and 110–120 in Bex4, and was predicted to be disordered. In Bex3 and Bex5, P residues were scattered throughout the N-terminal at residues 1–50 and 100–110, increasing the length of disordered region in the N-terminus to almost 50 residues. Third, a region rich in hydrophobic residues with a putative secondary structure was predicted close to the location of the coiled coils (residues 80–90 in all Bex proteins). In the case of Bex3 and Bex5 the long, horizontal cluster of hydrophobic residues clearly indicated the presence of the coiled coil. Finally, a region rich in R residues was predicted in all Bex proteins between residues 30 and 50. In Bex3 and Bex5 a second region rich in R residues was predicted at residues 70–80. Both R-rich regions flanked the predicted coiled coil. Interestingly, this R-rich region has been proposed to be necessary for nuclear shuttling between the cytosol and the nucleus and is located just before the predicted coiled-coil region [37, 40]. Based on this analysis we conclude that Bex proteins contain intrinsically disordered regions in the N-terminal (~50 residues in length) and the C-terminal (between residues 100 and 120), and a region comprising residues 70–100 containing putative secondary structure elements (α-helix and/or coiled coils).

In conclusion, our disorder predictions obtained using several servers, together with our HCA results, strongly suggest that Bex proteins are intrinsically disorder proteins (IDPs), and contain long disordered regions in the N- and C-terminals, with secondary structure elements in the region comprising residues 80–100. In some proteins (Bex3 and Bex5) this region adopts a coiled-coil structure.

### Predicted partner binding regions in Bex proteins

**Figure 5:**
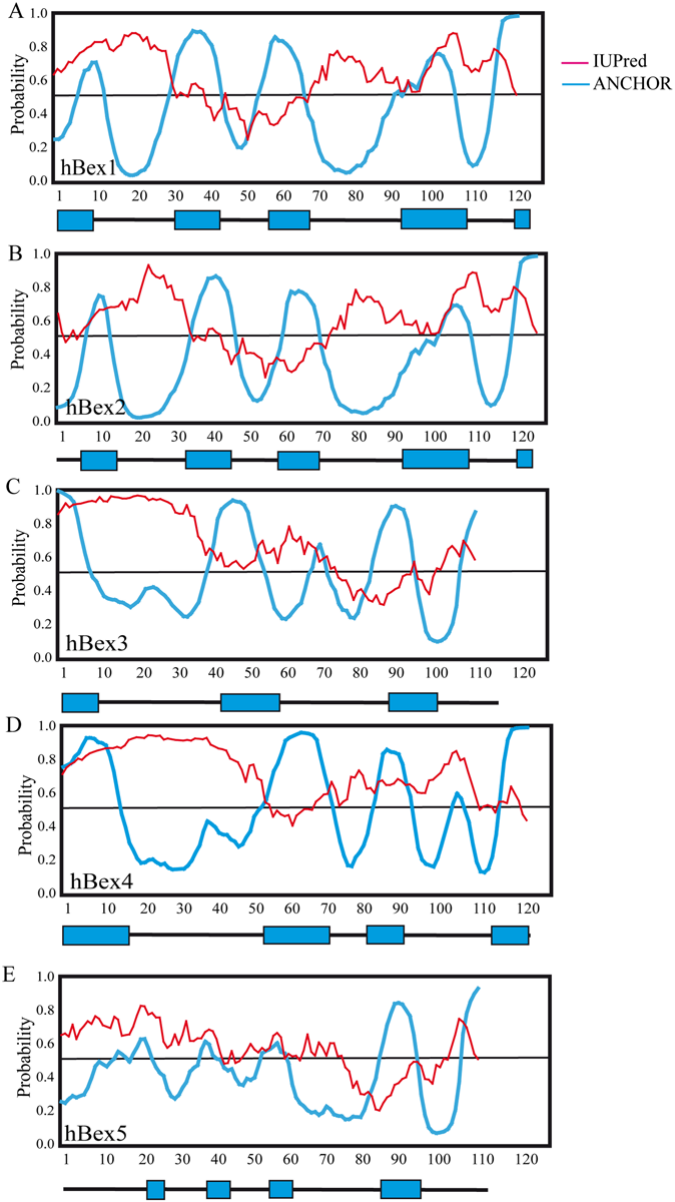
Locations of binding regions or Molecular Recognition Elements (MoRE) in Bex1–5 as predicted using ANCHOR. Red line denotes disordered regions predicted with IUPred and blue denotes the location of the predicted MoRE regions for A) hBex1, B) hBex2, C) hBex3, D) hBex4 and E) hBex5. The positions of the MoREs in each sequence are shown as blue rectangles. Green lines represent the location of the coiled coils predicted in Fig. 4.

Disorder-to-order transitions upon binding to partner proteins are mediated by small regions in IDPs called molecular recognition elements (MoREs). The presence of putative MoRE regions can be predicted in disordered regions using a variety of algorithms, such as ANCHOR [22, 49]. ANCHOR predicted the presence of five MoREs in the hBex1 protein sequence (Fig. 5A); two in the middle region, between residues K32 and E45 and residues V55 and M68, and another at the C-terminus between residues E92 and V108. These regions coincide with small cluster of hydrophobic residues in the HCA plot (Fig. 4). A very similar output was observed for hBex2 (Fig. 5B). Three MoREs were predicted in hBex3 (Fig. 4C): one in the N-terminal and two at residues 41–55 and 84–95. Interestingly, a MoRE located close to the putative coiled-coil domain described above was predicted for Bex1–3. Coiled coils are considered intrinsically disordered regions (IDRs), as they can undergo a disorder-to-order transition upon homodimerization or binding to a functional partner, but remain disordered in their free monomeric state.

**Figure 6:**
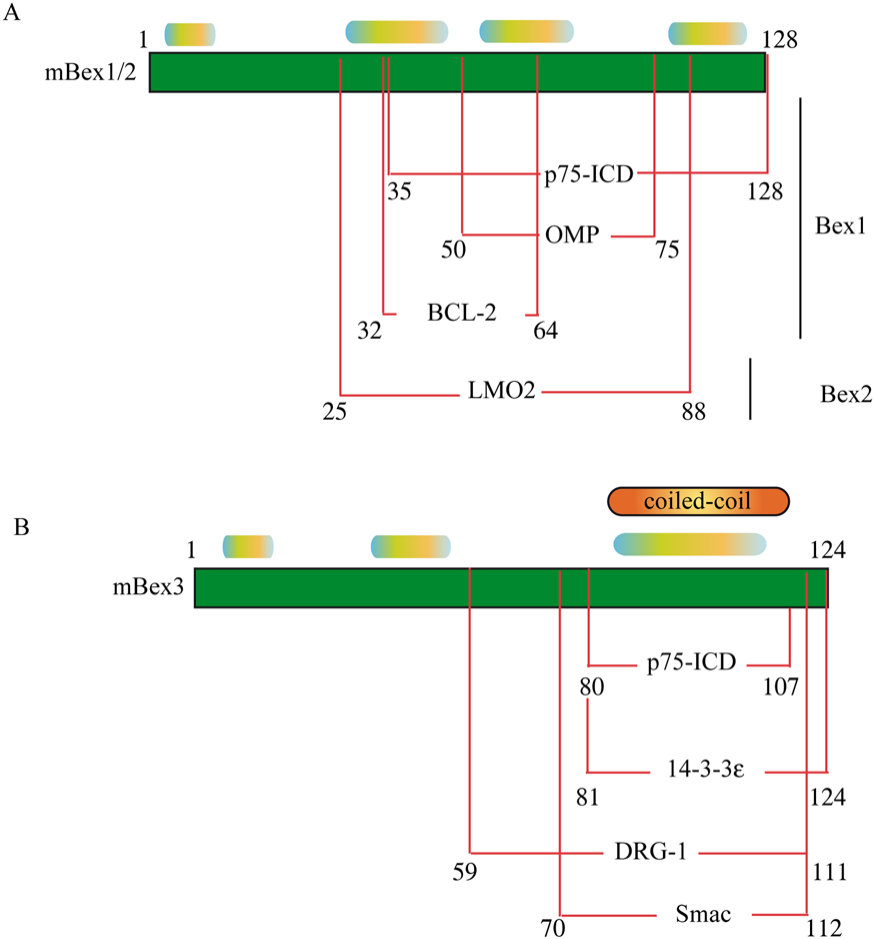
Summary of the regions that mediate interactions between Bex proteins and their intracellular partners. Regions in Bex1 and Bex2 (A) and Bex3 (B) that have been experimentally demonstrated to be necessary for such interactions are delimited by lines indicating the corresponding residues and intracellular partners. The predicted MoREs identified in this study are shown in yellow. For Bex3, the position of the coiled coil is indicated with an orange cylinder. References describing these interactions are cited in the main text.

The disordered regions in IDPs allow binding to multiple partners with low affinity and high specificity. We reviewed the published data on Bex binding partners to determine whether the MoREs predicted were of physiological significance. Experimentally, Bex proteins interact with a range of different proteins (Fig. 6). In several cases the mapping of these interactions has been reported, allowing us to compare the interaction region with our predicted MoREs (Fig. 6). Bex1 and Bex3 interact with the p75 intracellular domain (ICD). Bex1 interacts with p75ICD via residues 35–128 [9], a region that coincides with the location of some of the predicted MoREs (Fig. 5). Bex3 binds to p75ICD via residues 80–107 [43], which coincides with the predicted location of the MoRE shown in Fig. 5 and the coiled coil. Bex3 interacts with 14-3-3ε using the same region [44]. Bex1 and Bex3 also interact with other proteins. Bex1 binding to olfactory marker protein (OMP) is mediated by residues 50–75 [50], the same region in which one of our predicted MoREs was located. Although detailed structural analysis is lacking, the peptide derived from this region adopts an α-helix conformation upon binding to OMP, as has been shown by NMR [40, 50]. Bex3 interacts with DRG-1 [51] and Smac/Diablo [52] via its C-terminal region. While the Bex3 regions required for binding to TSC1 have not been defined, the coiled-coil region of TSC1 is required for this interaction [53]. Bex1 interacts with BCL-2 via residues 32–64 [54], the site of another of our predicted MoREs. Bex2 interacts with the transcription factor LMO2 via residues 25–88 [55], a less disordered region containing two putative MoREs. Taken together, these data experimentally confirm that our predicted MoRE regions are in fact true binding sites, and undergo disorder-to-order transitions upon binding. In the case of Bex3, the coiled-coil region clearly plays important functional roles in homo- or heterodimerization, as evidenced by the number of partners that use this region for binding. Bex3 may bind one partner via the coiled coil, leaving the other intrinsically disordered regions available for simultaneous interactions with one or more additional partners. The fact that disruption of Bex3 homodimerization impairs nuclear localization [45] suggests that that nuclear localization and nuclear shuttling are dependent on homodimerization.

### Bex1 is partially disordered in solution and adopts an α-helix structure upon TFE titration

**Figure 7:**
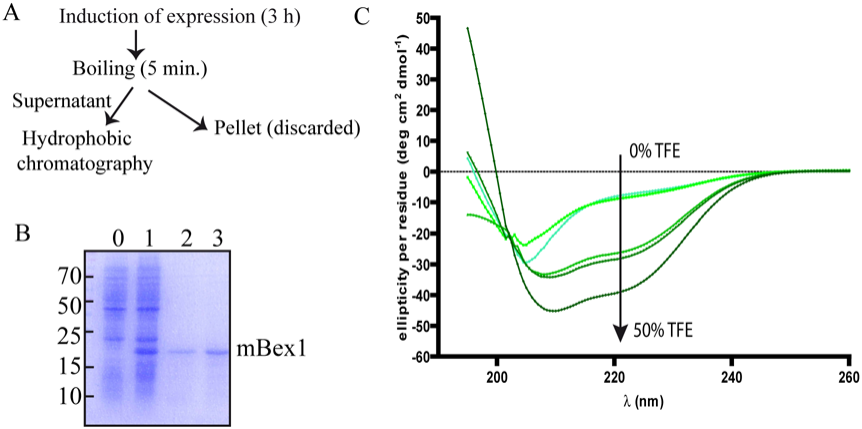
Analysis of mBex1 by far-UV circular dichroism (CD). A) Schematic showing the mBex1 purification process. After inducing protein expression with IPTG, pelleted bacteria were boiled. NaCl was added to the supernatant at a final concentration of 3 M and the solution was subjected to hydrophobic chromatography. B) SDS-PAGE of purified from *E. coli* by boiling and hydrophobic chromatography. Lane 0: no induction; lane 1: after incubation for 3 h with IPTG; lane 3: soluble fraction after boiling bacteria for 5 minutes; lane 4: eluent from hydrophobic chromatography. See text and Materials and Methods for details. Molecular weight standards are shown on the left. C) Far-UV CD spectra of mBex1 in 20 mM acetate buffer pH 5.1 containing increasing concentrations of trifluorethanol (TFE). The CD spectra shown represent the average of five independently determined spectra.

To confirm the internally disordered nature of Bex proteins, mouse Bex1 was expressed in bacteria and purified. Given that all Bex proteins share a similar percentage of disorder and a similar overall fold (disorder-order-disorder), we extrapolated our findings in Bex1 to the other members of the Bex family. Initially we attempted to purify Bex1 using GST and histidine tags at the N-terminus. Adequate purification was not achieved due to internal cleavage of the Bex1 protein and a low yield (data not shown). As Bex1 is a putative IDP we exploited the heat resistant properties of IDPs: we expressed full-length and un-tagged Bex1 in *E. coli*, and after inducing expression we boiled the bacterial pellet for 5 minutes (Fig. 7). SDS-PAGE analysis revealed the presence of almost 80% pure Bex1 in the supernatant (Fig. 6B). Next, we further purified Bex1 using hydrophobic interaction chromatography (HIC) (Fig. 7B). We selected this purification strategy, rather than classic size-exclusion chromatography (SEC), because the latter approach resulted in the elution of Bex1 bound to nucleic acids. The addition of a high salt concentration to the supernatant prior to hydrophobic chromatography countered the Bex1-nucleic acid interaction (see Methods for details of purification). After extensive dialysis Bex1 folding in solution was analyzed by circular dichroism (CD) spectroscopy. As shown in Fig. 6B, the CD spectrum of Bex1 in solution revealed a high level of disorder, although the observed spectrum did not exhibit a completely random coil conformation. Using the deconvolution software K2D3 [36], the percentage of α-helix was estimated at around 33% in solution, indicating that Bex1 contains secondary structure elements, probably in the form of nascent α-helices in some regions of the protein, as suggested by PSIPRED predictions (S1 Fig.). Trifluorethanol (TFE) has been used extensively as a stabilizing agent to reveal secondary structure elements by acting as a non-specific structure-inducer. In the case of IDPs, TFE reveals coil-to-helix transitions of disordered regions, suggesting that folding is induced in these regions upon binding to a physiological partner [56–58]. TFE could thus be used to elucidate IDP folding in the absence of their specific binding partners. Using CD spectroscopy we studied the changes in the secondary structure of Bex1 in response to increasing concentrations of TFE (Fig. 7C). Increasing concentrations of trifluorethanol (TFE) resulted in a higher α-helix percentage: K2D3 deconvolution software calculated a α-helix percentage of 87% at 50% TFE. Bex1 binds to the OMP protein, and previous published results demonstrated that this binding is mediated by the region in Bex1 comprising residues 70–80 [50]. Moreover, NMR analysis revealed that this region forms an α-helix upon binding [50]. While further detailed analysis of this region is necessary, these findings strongly suggest the presence of a nascent α-helix in this region of Bex1 that is stabilized upon partner interaction.

## Discussion

Although Bex1 was cloned in 1999, little is known about the structure of this protein. The *in silico* predictions and biochemical evidence presented here strongly support the inclusion of the Bex protein family in the growing class of intrinsically disordered proteins (IDPs).

**Figure 8:**
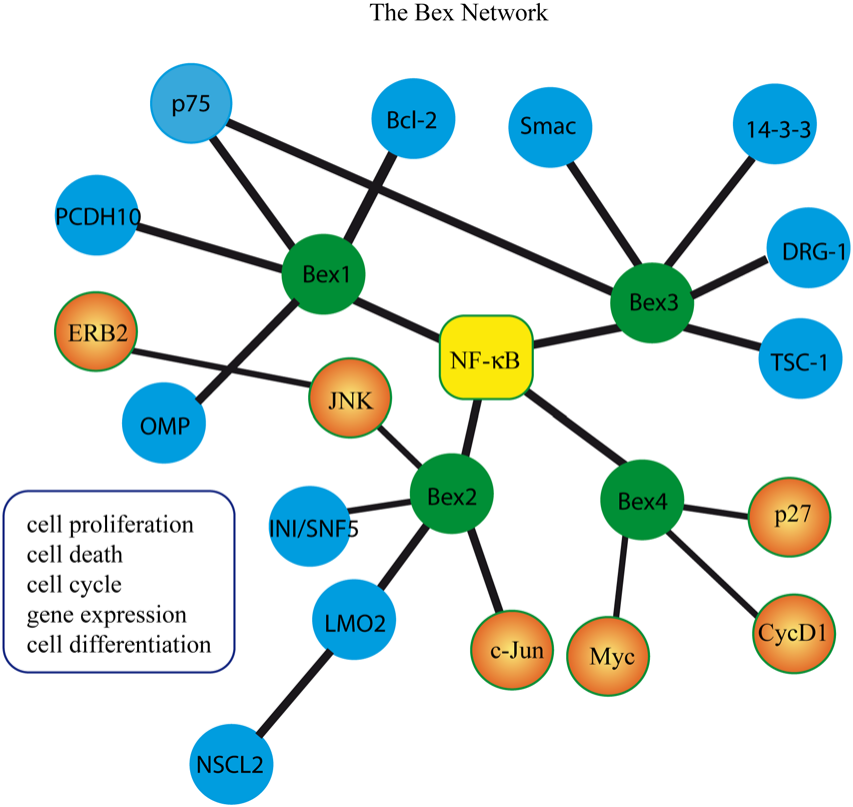
The Bex protein network. Bex proteins are promiscuous, interacting with a variety of proteins and participating in several signaling pathways. Bex1–4 are represented by green circles. Direct interactions described between Bex members and protein interactors are indicated with blue circles. Bex1–4 have been shown to modulate NF-kB signaling pathways, as indicated by the yellow square. Orange circles represent proteins whose expression or activation is modulated by the indicated Bex proteins.

IDPs have been implicated in a number of human diseases, including cancer, diabetes, and neurodegenerative and cardiovascular disorders. Abundant experimental evidence also supports a role of Bex proteins in cancer [4, 6, 37, 54, 59–69] and neurodegenerative diseases [70, 71]. Given their interactions with multiple partners and their intrinsically disordered nature, we propose that Bex proteins may form signaling hubs [72] (Fig. 8). Hubs formed by IDPs play important roles in cellular differentiation and cancer, and in transcriptional and translational regulation in both normal and pathological conditions [73–77]. In PC12 cells, Bex1 overexpression inhibits the induction of NF-κB activity by NGF without affecting the activation of Erk1/2 or AKT, while Bex1 downregulation increases neuronal differentiation and increases NF-κB activity in response to NGF [9]. Bex2 overexpression is associated with increased activation of the Bcl-2/NF-κB pathway in primary breast tumors [64, 67] and glioma cells [37]. Bex2 also regulates cell proliferation and apoptosis via a feedback loop between ErB2 and c-Jun [67, 68]. Bex1 and Bex3 (previously known as *p75NTR-associated cell death executor*, NADE) bind to the intracellular domain of p75 [9, 43], participate in p75(NTR)-induced signaling in the NF-κB pathway, and are implicated in cell death and the cell cycle [9, 43, 78]. Co-expression of Bex3 and p75NTR induces caspase-2 and caspase-3 activation and nuclear DNA fragmentation [43], and Bex3 appears to play a role in apoptosis through its interactions with Smac [52]. Downregulation of Bex4 (also known as *Transcription Elongation Factor S-II Protein-Like 7*, TCEAL7) results in increased NF-κB activity in ovarian cancer cells [79]. Several studies suggest that Bex proteins play regulatory roles in the transcriptional regulation of some genes and may form transcriptional hubs (Fig. 8). Bex2 binds to INI1/hSNF5, a key component of the SWI/SNF chromosome remodeling complex [80], and to the LIM_domain-containing transcription factor LMO2 [55]. Bex4 (TCEAL7) associates with cyclin D1 promoter containing Myc E-box sequence and transcriptionally represses cyclin D1 expression [81], and upregulates the promoter activity of the c-Myc-target gene, ornithine decarboxylase (ODC) [81]. Collectively, these data suggest that the NF-κB pathway lies at the center of the Bex protein network (Fig. 8) and constitutes a general signaling pathway in which Bex members play an important role.

In summary, we propose that the Bex protein family constitutes a bona-fide class of hub proteins which contain long disordered regions in addition to ordered regions (coiled coils), and can participate in multiple signaling pathways and in gene regulation. The inclusion of Bex proteins within the IDP group represents an important development in the field of Bex protein research, with important implications for future studies of the regulation and function of this interesting protein family.

## Supporting Information

S1 Fig Secondary structure prediction for hBex1 using PSIPRED from the PSIPRED server (http://bioinf.cs.ucl.ac.uk/psipred/). The secondary structure prediction is indicated with a pink helix (α-helix) or yellow arrow (β-sheet) above the protein sequence.
(TIF)

S2 Fig Secondary structure prediction for hBex2 using PSIPRED from the PSIPRED server (http://bioinf.cs.ucl.ac.uk/psipred/). The secondary structure prediction is indicated with a pink helix (α-helix) or yellow arrow (β-sheet) above the protein sequence.
(TIF)

S3 Fig Secondary structure prediction for hBex3 using PSIPRED from the PSIPRED server (http://bioinf.cs.ucl.ac.uk/psipred/). The secondary structure prediction is indicated with a pink helix (α-helix) or yellow arrow (β-sheet) above the protein sequence.
(TIF)

S4 Fig Secondary structure prediction for hBex4 using PSIPRED from the PSIPRED server (http://bioinf.cs.ucl.ac.uk/psipred/). The secondary structure prediction is indicated with a pink helix (α-helix) or yellow arrow (β-sheet) above the protein sequence.
(TIF)

S5 Fig Secondary structure prediction for hBex5 using PSIPRED from the PSIPRED server (http://bioinf.cs.ucl.ac.uk/psipred/). The secondary structure prediction is indicated with a pink helix (α-helix) or yellow arrow (β-sheet) above the protein sequence.
(TIF)
