# Supplementary figures and images for: Brain Expressed and X-Linked (Bex) Proteins Are Intrinsically Disordered Proteins (IDPs) and Form New Signaling Hubs
Source: PLoS One. 2015 Jan 22;10(1):e0117206. doi: 10.1371/journal.pone.0117206 (PMC4303428; doi:10.1371/journal.pone.0117206)

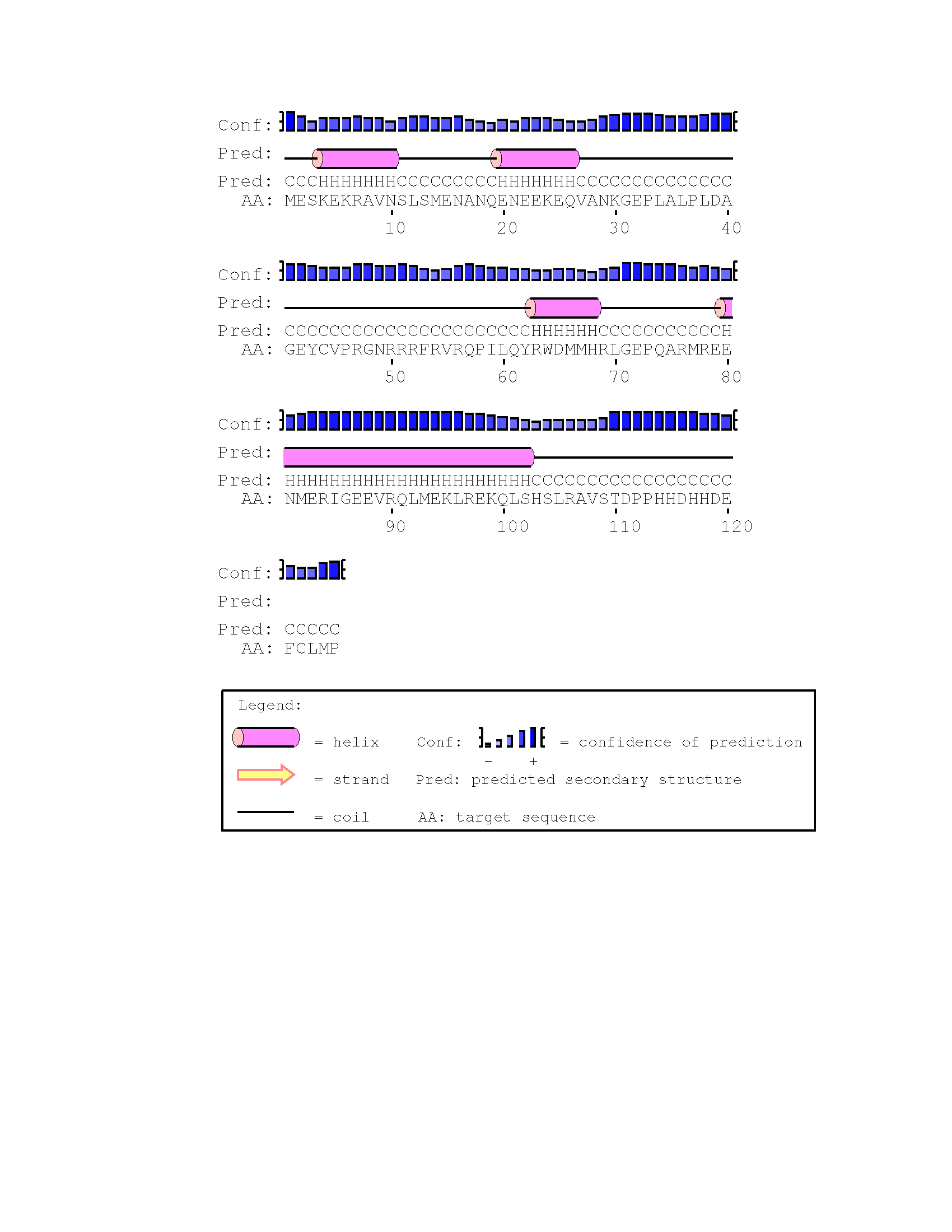

Supplement: S1 Fig — The secondary structure prediction is indicated with a pink helix (α-helix) or yellow arrow (β-sheet) above the protein sequence. (TIF) [file pone.0117206.s001.tif]

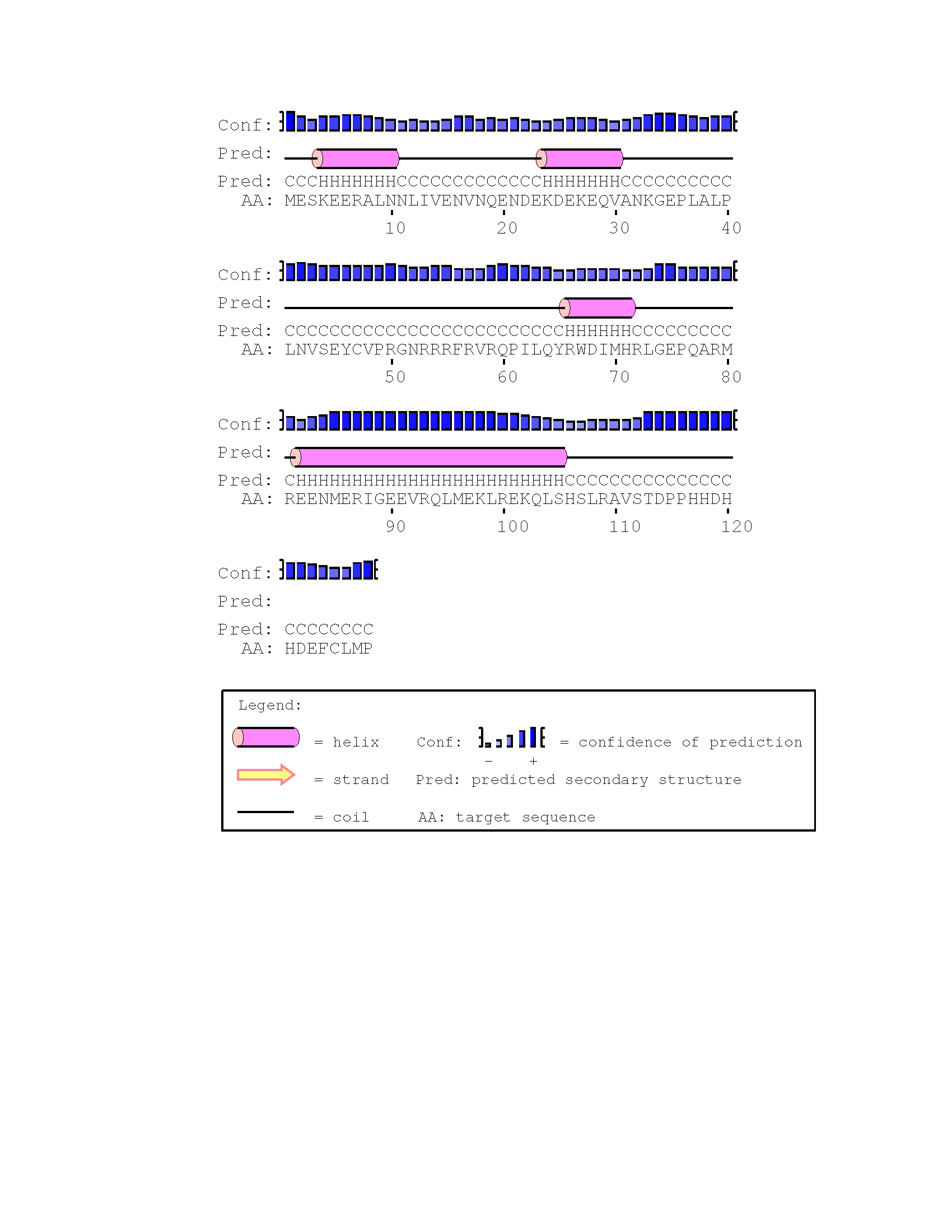

Supplement: S2 Fig — The secondary structure prediction is indicated with a pink helix (α-helix) or yellow arrow (β-sheet) above the protein sequence. (TIF) [file pone.0117206.s002.tif]

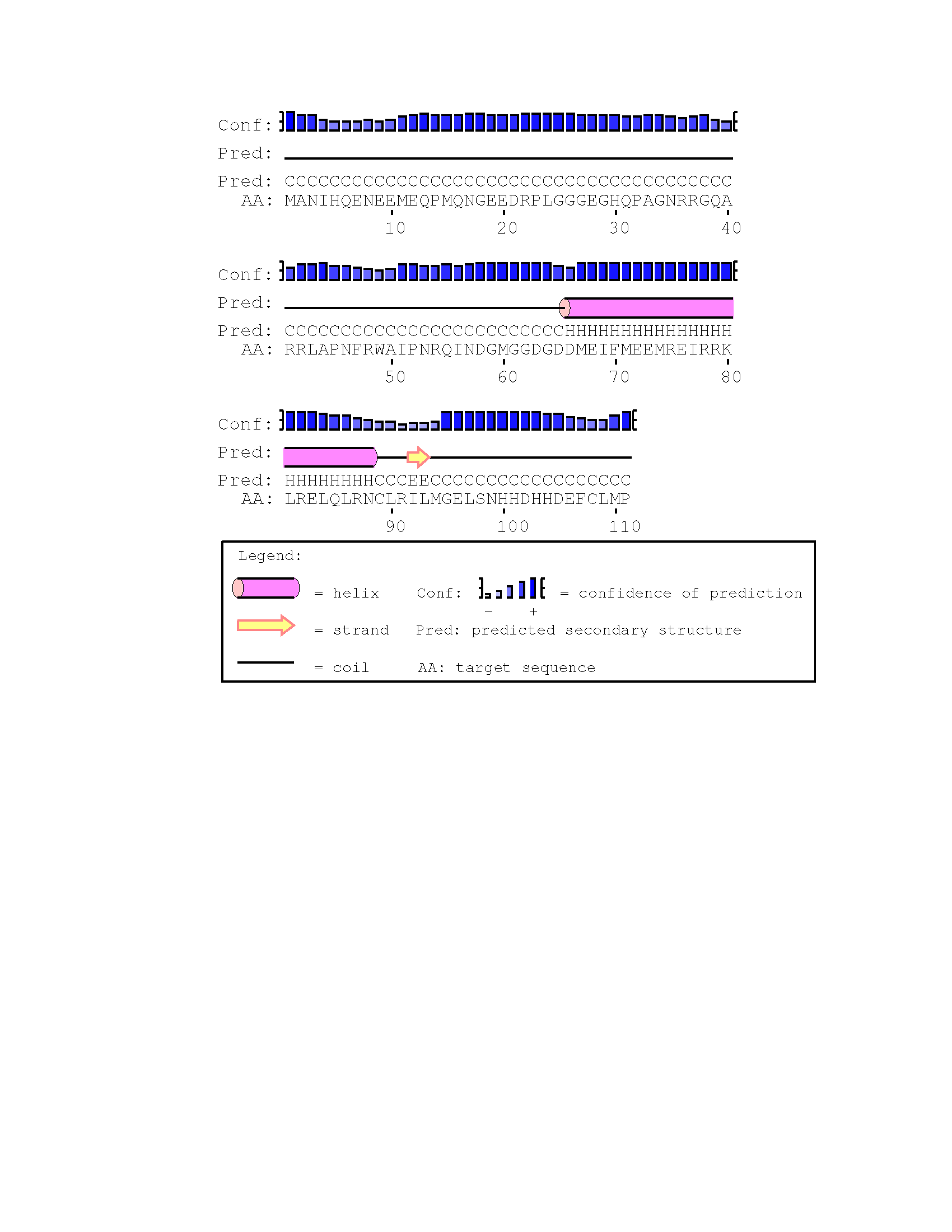

Supplement: S3 Fig — The secondary structure prediction is indicated with a pink helix (α-helix) or yellow arrow (β-sheet) above the protein sequence. (TIF) [file pone.0117206.s003.tif]

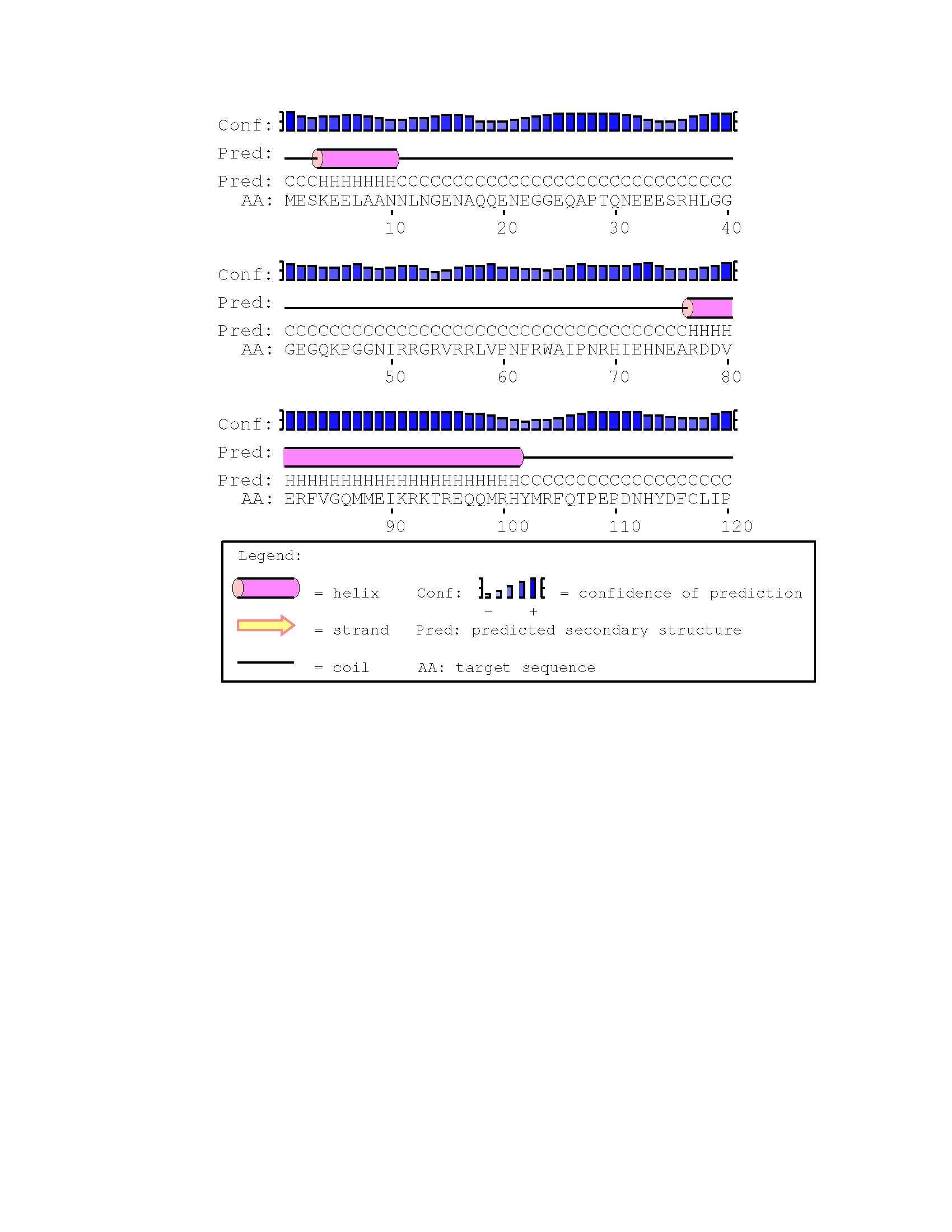

Supplement: S4 Fig — The secondary structure prediction is indicated with a pink helix (α-helix) or yellow arrow (β-sheet) above the protein sequence. (TIF) [file pone.0117206.s004.tif]

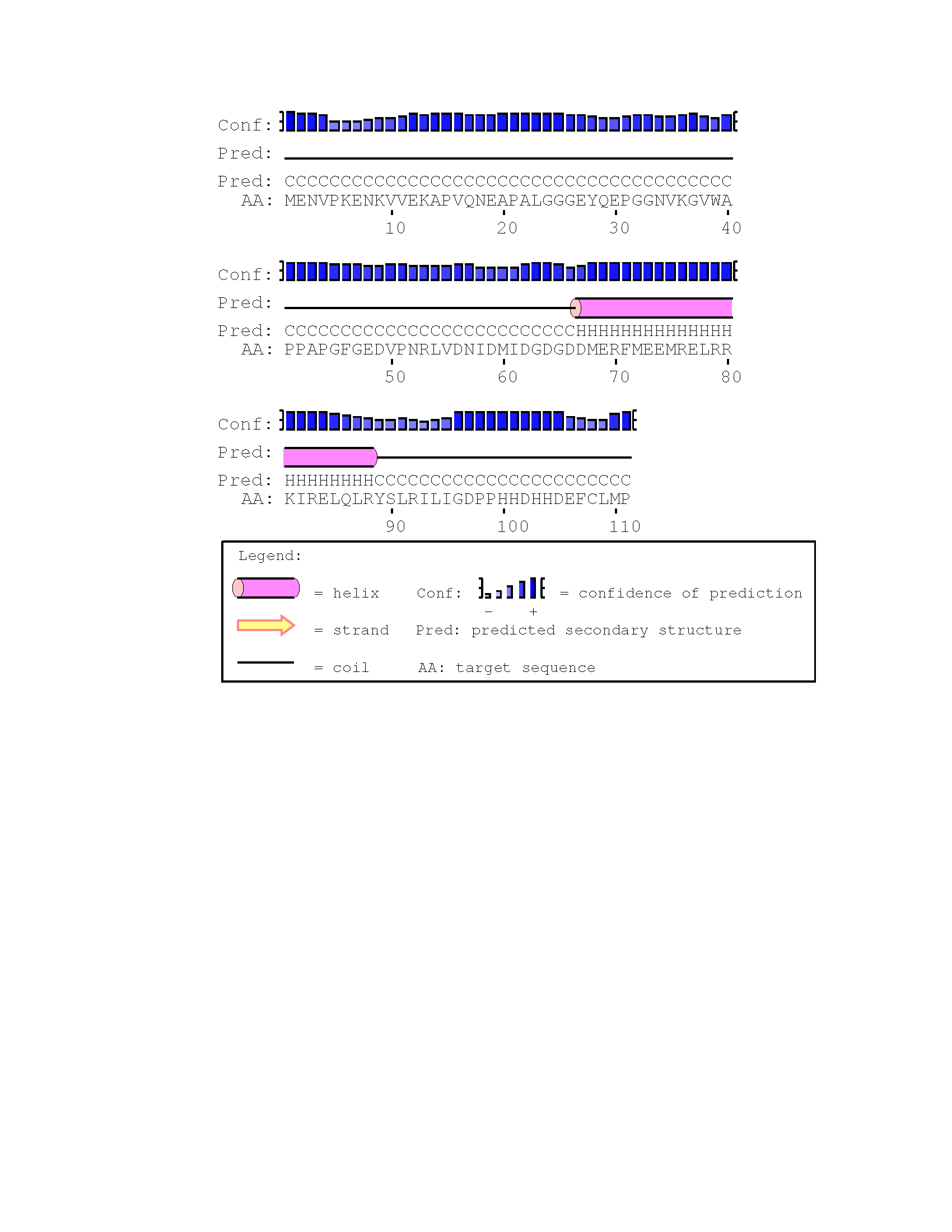

Supplement: S5 Fig — The secondary structure prediction is indicated with a pink helix (α-helix) or yellow arrow (β-sheet) above the protein sequence. (TIF) [file pone.0117206.s005.tif]
